# Draft Genome Sequence of Bacillus velezensis B6, a Rhizobacterium That Can Control Plant Diseases

**DOI:** 10.1128/genomeA.00182-18

**Published:** 2018-03-22

**Authors:** Yu-han Gao, Rong-jun Guo, Shi-dong Li

**Affiliations:** a Institute of Plant Protection, Chinese Academy of Agricultural Sciences, Beijing, China

## Abstract

The draft genome of Bacillus velezensis strain B6, a rhizobacterium with good biocontrol performance isolated from soil in China, was sequenced. The assembly comprises 32 scaffolds with a total size of 3.88 Mb. Gene clusters coding either ribosomally encoded bacteriocins or nonribosomally encoded antimicrobial polyketides and lipopeptides in the genome may contribute to plant disease control.

## GENOME ANNOUNCEMENT

Bacillus velezensis strain B6, which was previously reported as Bacillus subtilis (1), was isolated from soil in China. Through successful colonization in rhizosphere and the production of antifungal metabolites, this strain performs good biocontrol efficacy in suppressing cucumber wilt disease caused by Fusarium oxysporum and pepper root rot caused by Phytophthora capsici. In addition, B. velezensis B6 could inhibit the multiplication and delay the spread of F. oxysporum in cucumber stems (2, 3) to suppress the disease development. However, the genes related to the biocontrol functions of B. velezensis B6 are not well known. Here, we report the draft genome sequence of B. velezensis B6.

Whole-genome sequencing was carried out by BGI Tech Solutions Co., Ltd. (People’s Republic of China) with the Illumina HiSeq 2000 platform. A total of 586 Mb of data was generated for the genome of B. velezensis B6. The short reads were assembled into genome sequences by using SOAPdenovo 2.04 (4, 5). The draft genome sequence of strain B6 comprises 32 scaffolds, with a total size of 3,879,012 bp and a GC content of 46.55%.

With SOAPaligner 2.21 sequence alignment software, all read sequences obtained by sequencing were aligned with the reference genome sequence of B. velezensis FZB42 (previously Bacillus amyloliquefaciens subsp. plantarum FZB42) (6). Indels and single nucleotide polymorphisms (SNPs) were detected based on the alignment between the assembly results and reference genome. The indel analysis results exhibited 243 insertion mutations and 249 deletion mutations, and 84 indels located in the coding regions of the genomic DNA sequence. A total of 38,320 SNPs were present in the genome of strain B6, including 8,917 nonsynonymous mutations. Some nonsynonymous mutations occurred at functional genes, such as the surfactin-encoding genes *srfAA*, *srfAB*, *srfAC*, and *srfAD*, the competence protein S gene *comS*, and the spore germination protein genes *gerKA*, *gerKB*, and *gerKC*. Whether these mutations lead to functional variations should be further investigated.

The annotation predicted gene clusters coding either ribosomally encoded bacteriocins or nonribosomally encoded antimicrobial polyketides and lipopeptides contained in the genome of strain B6. These metabolites are antimicrobial agents with multiple functions. The encoding gene of mycosubtilin, the most active form in the iturin family (7), was found in the strain B6 genome. Mycosubtilin enhances spreading of Bacillus subtilis (8) and exhibits antibacterial, antifungal, and strong hemolytic activities (9). Strain B6 also harbors the encoding genes of surfactin and plipastatin (fengycin) (10), which show strong antibacterial and antifungal activities, respectively (11). In addition to these lipopeptide antibiotics, strain B6 also contains genes to synthesize the catechol-type siderophore bacillibactin (12) and the antibiotics bacillaene (13) and bacilysin (14). The capability to produce these potent antimicrobial compounds may contribute to the ability of B. velezensis B6 to control plant diseases.

### Accession number(s).

This whole-genome shotgun project has been deposited at DDBJ/ENA/GenBank under the accession number NEOS00000000. The version described in this paper is version NEOS01000000.
